# Microenvironment commits breast tumor ECs to dedifferentiation by micro-RNA-200-b-3p regulation and extracellular matrix remodeling

**DOI:** 10.3389/fcell.2023.1125077

**Published:** 2023-05-16

**Authors:** Kinga Wilkus-Adamczyk, Klaudia Brodaczewska, Aleksandra Majewska, Claudine Kieda

**Affiliations:** ^1^ Laboratory of Molecular Oncology and Innovative Therapies, Military Institute of Medicine- National Research Institute, Warsaw, Poland; ^2^ Postgraduate School of Molecular Medicine, Medical University of Warsaw, Warsaw, Poland; ^3^ Center for Molecular Biophysics UPR 4301 CNRS, Orleans, France

**Keywords:** breast cancer, endothelial cells (ECs), ECM-receptor interaction (extracellular matrix-receptor interaction), endothelial to mesenchymal transition (EndMT), heterogeneity, miR-200b-3p

## Abstract

**Introduction:** Hypoxia shapes the tumor microenvironment, modulates distinct cell population activities, and activates pathological angiogenesis in cancer, where endothelial cells (ECs) are the most important players. This study aimed to evidence the influences of the tumor microenvironment on the global gene expression pattern characteristic for ECs and the distinct responses displayed by tumor-derived ECs in comparison to the healthy endothelium during endothelial to mesenchymal transition (EndMT) and its regulation by miR-200-b-3p.

**Methodology:** Immortalized lines of ECs from the same patient with breast cancer, healthy breast tissue (HBH.MEC), and primary tumor (HBCa.MEC) were used. The experiments were performed in normoxia and hypoxia for 48 h. By using the wound healing test, we investigated the migration abilities of ECs. Global gene expression analysis with NGS was carried out to detect new pathways altered in pathological ECs and find the most changed miRNAs. The validation of NGS data from RNA and miRNA was estimated by qPCRs. Mimic miR-200b-3p was used in HBH.MEC, and the targets *VEGF*, *Bcl2*, *ROCK2*, and *SP1* were checked.

**Results:** Hypoxia influences EC migration properties in wound healing assays. In hypoxia, healthy ECs migrate slower than they do in normoxia, as opposed to HBCa.MEC, where no decreased migration ability is induced by hypoxia due to EndMT features. NGS data identified this process to be altered in cancer ECs through extracellular matrix (ECM) organization. The deregulated genes, validated by qPCR, included *SPP1*, *ITGB6*, *COL4A4*, *ADAMST2*, *LAMA1*, *GAS6*, *PECAM1*, *ELN*, *FBLN2*, *COL6A3*, and *COL9A3*. NGS also identified collagens, laminins, fibronectins, and integrins, as being deregulated in tumor-derived ECs. Moreover, the analysis of the 10 most intensively modified miRNAs, when breast tumor–derived ECs were compared to healthy ECs, shed light on miR-200b-3p, which is strongly upregulated in HBCa.MECs when compared to HBH.MECs.

**Discussion and conclusion:** The pathological ECs differed significantly, both phenotypically and functionally, from the normal corresponding tissue, thus influencing their microenvironment cross-talk. The gene expression profile confirms the EndMT phenotype of tumor-derived ECs and migratory properties acquisition. Moreover, it indicates the role of miR-200b-3p, that is, regulating EndMT in pathological ECs and silencing several angiogenic growth factors and their receptors by directly targeting their mRNA transcripts.

## 1 Introduction

The tumor microenvironment contributes not only to the diversity of tumor blood vessels in tissues but also to endothelial cell heterogeneity. These cells are the key players in pathological angiogenesis driven by hypoxia, defined as lower than physiological oxygen tension. Aside from angiogenesis, hypoxia influences ECs proliferation, migration, and deep phenotypic changes up to the induction of endothelial to mesenchymal transition (EndMT) in the microenvironment of hypoxia-dependent pathologies (Carreau et al., 2011). In this process, endothelial cells (ECs) present a reduced expression of typical endothelial markers, which include vascular endothelial cadherin (VE-cadherin), platelet endothelial cell adhesion molecule (PECAM-1/CD31), and von Willebrand Factor (vWF) (Kim, 2018). ECs gain a few mesenchymal cell features such as loss of tight junctions, increased motility, and increased level of extracellular matrix proteins such as collagens, elastin, fibronectins, and laminins (Kim, 2018). This process prompts cancer propagation and metastasis; thus, it is crucial to understand the crosstalk between endothelial cells and their microenvironment and how it is different in biologically healthy and pathological tissues. This is especially significant because the degree of susceptibility of ECs to selective interactions depends on their organ/tissue specificity on the one hand and biological commitment to the concerned disease on the other.

A major component of the microenvironment is the extracellular matrix (ECM), which is a set of extracellular molecules that provide structural and biochemical support for cells. The ECM plays an important role in cell adhesion, migration, intercellular communication, and differentiation (Provenzano et al., 2009). It is composed of collagens, proteoglycans (PGs), glycosaminoglycans (GAGs), elastin, laminins, and fibronectins (Theocharis et al., 2016). The ECM constitutes the scaffold for the organizational maintenance of vascular ECs in blood vessels, mostly through adhesive interactions with integrins on the endothelial cell surface. Moreover, adhesion of ECs to the ECM is required for their proliferation, migration, neovascularization, and blood vessel maturation (Davis and Senger, 2005). The structure of the ECM that allows ECs to contact their close environment through angiocrine signaling consists of a basement membrane (BM), which is composed predominantly of laminins and collagen IV strands. Collagen IV and laminins provide structural stability and transducing signals that control cell migration, survival, proliferation, and differentiation (Miner, 2008). Laminins take part in adhesion, differentiation, migration, phenotype maintenance, and apoptotic resistance (Liu et al., 2008). Through binding of integrins, laminins create a link between the cell and its ECM. The glycocalyx is largely composed of membrane‐bound proteoglycans (GAGs), sequesters growth factors, and functions as a signaling platform. In addition, the ECM provides informational cues to the vascular cells, regulating their proliferation and differentiation (Zhang et al., 2005; Caiado et al., 2011). This occurs both physiologically during wound healing and pathologically during tumor growth; the sprouting of new blood vessels during angiogenesis is the fundamental step for angiogenesis, in which the ECM plays a key role (Theocharis et al., 2016). In addition to pathological angiogenesis, the surrounding ECM undergoes changes in a dynamic interplay between the microenvironment and resident cells during tumor cell proliferation. These changes, which include increased secretion of fibronectin and collagens I, III, and IV, illustrate that tumor progression demands a continuous interaction between the ECM and tumor cells (Malik et al., 2015). Furthermore, activated matrix metalloproteinases (MMPs) are involved in the degradation of the extracellular matrix, which contributes to the pathogenesis of cancer (Niland et al., 2021). ADAMTS2 is an example of an MMPs family member that suppresses tumor growth by inhibiting angiogenesis (Kumar et al., 2012). Another MMP that plays an important role in endothelial cell migration, a key feature of angiogenesis, is matrix metalloproteinase-2 (MMP-2) (Mook et al., 2004). It has been shown that tissue inhibitors of matrix metalloproteinase 2 (TIMP2) negatively regulate endothelial cell migration and invasion in many cancers (Brew et al., 2000).

In addition to ECM gene expression changes, EndMT is regulated by microRNAs (miRNAs), which are short (∼22 nucleotides) RNA molecules transcribed from noncoding genes that regulate post-transcriptionally the gene expression at the mRNA level by binding to their 3′-untranslated region (3′-UTR), inhibiting the translation or causing mRNA degradation (Zhao et al., 2021). miRNAs are involved in pathological processes, such as tumorigenesis (Dalmay, 2013). It has been widely shown that dysregulation of miRNAs is associated with the initiation, development, progression of cancer, and dedifferentiation (Cho, 2012). Several miRNAs are described to regulate EndMT, as reviewed by Kim (2018). A few of them, such as miR-200b-3p, have been shown to inhibit EndMT by directly targeting the transcription factors or inhibiting signaling pathways associated with the induction of EndMT (Kim, 2018). The family of miR-200 includes five members: miR-200a, miR-200b, miR-200c, miR-429, and miR-141 (Zidar et al., 2016). miR-200b-3p, along with other miR-200 family members, is considered to be antioncogenic due to its suppressive effects in most tumors. It works especially in inhibiting tumor cell proliferation, apoptosis, invasion, and migration. In breast cancer, it has been demonstrated that miR-200b inhibits the epithelial to mesenchymal transition (EMT) by hampering the transcription factors' activity (Gregory et al., 2008). It has been proven that miR-200b can repress angiogenesis by targeting the synthesis of angiogenic factors and receptors in breast tumor tissue (Chang et al., 2013). Very little is known about the regulation of miR-200b-3p in breast tumor–derived ECs. Thus, it is of pivotal importance to understand whether the ECs' crosstalk and reactivity are triggered as a function of their origin, and more importantly, how the pathologically modified endothelial cells reflect the disease and participate in its propagation.

This work attempts to clarify, by the designed endothelial cells model, the distinct properties that the endothelium can display in setting wound healing/angiogenesis in pathological sites when compared to healthy microenvironments. This should shed light on potential new strategies to address treatments that can modulate the microenvironment in order to better target tumor cells. Such EC-relative study at the molecular level allows deciphering the causes that make pathologic angiogenesis distinct from the normally achieved process of vessel formation.

## 2 Methodology

### 2.1 Endothelial cell line culture

This study uses cell lines previously characterized, which were obtained as a part of our previous study that aimed to establish EC lines representative of distinct tissues to demonstrate their organ specificity (Kieda et al., 2002; Wilkus et al., 2021). The fresh specimens of breast tissues were gifted by Prof. Salem Chouaib (INSERM UMR 1186, Gustave Roussy Institute, Villejuif, France). Samples were obtained during standard tumor resections; patient information was de-identified and tissues were processed in the laboratory as described previously (CNRS patent 113 99-16169) (Wilkus et al., 2021). Cell lines named HBH.MEC and HBCa.MEC represented endothelial cells of healthy tissue and primary breast tumor, respectively, and were deposited in the National Collection of Cells and Microorganisms (Pasteur Institute). Both cell lines were seeded at a density of 5 × 10^4^ cells/10 cm^2^ on Primaria Tissue Culture Flask (Corning, NY, United States, #353808) in the presence of Opti-MEM I Reduced Serum Medium (Gibco, Paisley, United Kingdom #31985070) supplemented with 2% (vol/vol) fetal bovine serum (Gibco, Paisley, United Kingdom #A3840402). The cell lines were maintained at 37°C with 5% CO_2_ in a humidified atmosphere. After cell culture in the described conditions, the cells were detached using Accutase supplied in Dulbecco’s PBS containing 0.5 mM EDTA and phenol red (Invitrogen, Carlsbad, CA, United States #E136579). Additionally, all the cells were negative for *Mycoplasma* (PromoKine, Heidelberg, Germany #PK-CA91-1096).

### 2.2 Normoxic and hypoxic culture conditions

HBH.MEC and HBCa.MEC were seeded and cultured in normoxia (∼19% O_2_) for 8 h as described above. Next, the medium was changed to normoxic or hypoxic (stored for 24 h in suitable conditions) balanced medium, and the cells were cultured in standard conditions (normoxia) or transferred to hypoxia (pO_2_ = 1%) chamber (BioSpherix, Xvivo System Model X3, United States). Hypoxic conditions were maintained by the gas exchange with 5% CO_2_ and 94% N_2_ mix. The cells were cultured for 48 h in each condition. The sub-confluent monolayers of ECs were used for the desired tests and treatments.

### 2.3 Cell migration assay

The migration of both EC lines was investigated using the wound healing method. The cells were seeded onto 24-well plates, incubated with complete medium at 37°C with 5% CO_2_ overnight. Thereafter, a scratch was made and fresh medium, normoxic or hypoxic, was added to the cells. Then, the plates with both cell lines were put under normoxia or hypoxia. The cells were observed and photographed at specific times (0, 6, 12, 18, and 24 h) under ×5 magnification using the Z1/7 software Zen v.2.6 (Zeiss, Oberkochen, Germany). The distances between the edges of the scratch were measured using the ImageJ software (v.1.52p).

### 2.4 Protein secretion

TIMP-2 and MMP-2 were measured by using the bead-based immunoassay LEGENDplex (BioLegend, United States). Conditioned medium (CM) was collected from the cells cultured for 48 h, centrifuged at 500×*g* for 10 min to remove cells and frozen. CM samples were thawed on ice and incubated with capture beads for 2 h in RT with shaking. Then, washed twice and incubated with detection antibodies for 1 h in RT with shaking. Detection was performed by SA-PE incubation for 30 min in RT with shaking and then analyzed by flow cytometry using the CYTOFLEX software v.2.3.0.84 (Beckman Coulter, United States) according to the manufacturer’s instructions. The range of detection was tested by measuring recombinant proteins provided in the kit.

### 2.5 Next-generation sequencing: RNA based

After the total RNA isolation using the RNeasy Mini Kit (QIAGEN, Hilden, Germany, #74136) according to the manufacturer’s protocol, the concentration of the precipitates was measured using the RNA BR Assay Kit (Thermo Fisher Scientific, Singapore, #10210). The quality and integrity of the material were evaluated using the Qubit RNA IQ Assay Kit (Thermo Fisher Scientific, Singapore, #33221). Samples with IQ >8.5 were used for sequencing. Libraries were purified with the NEBNext Sample Purification Beads (New England Biolabs, Ipswich, MA, United States, #E7770S) and their quality was confirmed by using the High Sensitivity DNA Kit and Bioanalyzer (Agilent Technologies, Santa Clara, CA, United States, #5067-4626). Sequencing was performed by the external service Lexogen GmbH (Vienna, Austria) using the NextSeq 500 System (Illumina, San Diego, CA, United States). Differentially expressed genes (DEGs) were identified by −1.5 < logFC > 1.5 and *p*-value < 0.5. Gene symbols were translated into UniProt accession numbers using the UniProt Knowledgebase (UniProtKB). Protein networks were constructed in the STRING (Search Tool for the Retrieval of Interacting Genes) database using the list of protein accession numbers as a query and then analyzed with the Cytoscape software (v.3.8.0) to identify Gene Ontology (GO) biological processes and the Kyoto Encyclopedia of Genes and Genomes (KEGG) pathways represented by the DEGs with statistical significance.

### 2.6 Quantitative real-time PCR RNA

First, total RNA extraction using the RNeasy Mini Kit (QIAGEN, Hilden, Germany, #74136) in accordance with the manufacturer’s protocol was performed. The relative mRNA level was calculated using the 2^–∆∆Ct^ method, with normalization to the expressions of *YWHAZ* and *PPIA.* The sequences of the primers used in the reactions are given in Table 1.

**TABLE 1:** List of primer sequences used for the gene expression assessment.

Gene	Sequence
SPP1_FORhu	CAA​ACG​CCG​ACC​AAG​GAA​AA
SPP1_REVhu	GGC​CAC​AGC​ATC​TGG​GTA​TT
ITGB6_FORhu	GGG​GAT​TGA​ACT​GCT​TTG​CC
ITGB6_REVhu	AGG​GCA​CAG​CCA​CCT​TGT​A
COL4A4_FORhu	CCCGCTTGTTCCCCGC
COL4A4_REVhu	TGG​GTC​AAA​GTC​TGT​TCC​TGT
ADAMST2_FORhu	CCG​CTA​CCT​GCA​CTC​CTA​TG
ADAMST2_REVhu	GCA​CAC​ATA​GTC​CCG​TCC​AA
LAMA1_FORhu	TGC​TTC​TGC​TTT​GGC​GTT​TC
LAMA1_REVhu	GCC​AAA​CGC​AGT​CAG​CTT​AT
GAS6_FORhu	CGA​CCC​CGA​GAC​GGA​TTA​TT
GAS6_REVhu	GGC​GAA​GCC​TGA​GTT​TTT​GG
PECAM1_FORhu	GAC​GTG​CAG​TAC​ACG​GAA​GT
PECAM1_REVhu	GGA​GCC​TTC​CGT​TCT​AGA​GTA​T
ELN_FORhu	AGGTGCGGTGGTTCCTCA
ELN_REVhu	CAA​AGG​GTC​CAA​CTC​CTG​GG
FBLN2_FORhu	GCCCCGCGGGTCTTAC
FBLN2_REVhu	CGC​CTC​CTC​AAT​GCA​GTT​CT
COL6A3_FORhu	CAG​GTT​TGC​TCA​GGG​GTT​CA
COL6A3_REVhu	ATA​TCA​GCA​GCC​GCA​CCA​TT
COL9A3_FORhu	CGG​ACC​CAA​AGG​AGA​GTC​TGG
COL9A3_REVhu	GCG​CGG​CTA​ACT​GTG​CAA​T
COL9A3_REVhu	GCG​CGG​CTA​ACT​GTG​CAA​T
VEGF A_FORhu	TTG​CCT​TGC​TGC​TCT​ACC​TCC​A
VEGF A_REVhu	GAT​GGC​AGT​AGC​TGC​GCT​GAT​A
ROCK2_FORhu	TGC​GGT​CAC​AAC​TCC​AAG​CCT​T
ROCK2_REVhu	CGT​ACA​GGC​AAT​GAA​AGC​CAT​CC
SP1_FORhu	TTG​CTG​CTA​TGC​CAA​ACC​TA
SP1_REVhu	CCT​GAG​AGC​TGG​GAG​TCA​AG
Bcl2_FORhu	CAG​GAT​AAC​GGA​GGC​TGG​GAT​G
Bcl2_REVhu	GAC​TTC​ACT​TGT​GGC​CCA​GAT
PPIA_FORhu	CCG​CGT​CTC​CTT​TGA​GGT​AA
PPIA_REVhu	GCT​GCA​CGA​TCA​GGG​GTA​A
YWHAZ_FORhu	TGA​TCC​CCA​ATG​CTT​CAC​AAG
YWHAZ_REVhu	GCC​AAG​TAA​CGG​TAG​TAA​TCT​CC

### 2.7 Next-generation sequencing and bioinformatic analysis of miRNAs

First, RNA concentration and integrity were measured with UV-Vis spectrophotometry (NanoDrop 2000c, Thermo Fisher Scientific) and the Fragment Analyzer system using the DNF-471 RNA Kit (15 nt) (Agilent, Santa Clara, CA, United States), respectively. As per the manufacturer’s protocol, the libraries were generated using 100 ng RNA samples with the Small RNA-Seq Library Prep Kit (Lexogen, Vienna, Austria, #052UG128V0110). Then, the cDNA library with adapters for sequencing was prepared and pooled in an equimolar ratio. The concentration and size distribution of the final lane mix was analyzed by using the Qubit dsDNA HS assay (Thermo Fisher Scientific, Waltham, MA, United States) and the Fragment Analyzer system using the DNF-474 HS NGS Fragment Kit (1–6,000 bp) (Agilent, Santa Clara, CA, United States). Dilution of the lane mix (2 nm) was denatured and diluted to loading concentration for sequencing in the NextSeq 500 instrument using the SR75 High Output Kit (Illumina). Differentially expressed miRNAs (DEmiRNAs) were classified according to −1 < logFC > 1 and adjusted *p*-value and FDR (false discovery rate) <0.05.

### 2.8 RNA extraction, cDNA synthesis, and quantitative real-time PCR miRNAs

Total RNA from the endothelial cell line samples was isolated as provided in the abovementioned experiments (QIAGEN, Hilden, Germany, #74136), and reverse transcription reaction was performed according to the manufacturer’s recommendations using the TaqMan™ Advanced miRNA cDNA Synthesis Kit (Applied Biosystems, MS, United States, #A28007). Quantitative real-time polymerase chain reaction (qPCR) was performed using the TaqMan™ Fast Advanced Master Mix and TaqMan™ Advanced miRNA Assays (Applied Biosystems, Carlsbad, CA 92008 United States) (Table 2). Relative miR quantities were estimated using the comparative threshold cycle (∆Ct) and calculated using the 2^−ΔΔCT^ method. Different samples were normalized to miR-16-5p and miR-25-3p expressions.

**TABLE 2:** List of TaqMan probes used to detect miRNA expression.

miRNA	Assay number
miR-1271-5p	478674_mir
miR-222-3p	477982_mir
miR-200b-3p	477963_mir
miR-199b-5p	478486_mir

### 2.9 Functional analysis of miR-200b-3p in HBH.MEC

HBH.MEC cells were transfected with 10 nM mirVana hsa-miR-200 Mimic (#4464066, Thermo Fisher Scientific) using Lipofectamine RNAiMAX transfection reagent (#13778075, Thermo Fisher Scientific) according to the manufacturer’s instructions. As a negative control transfection with the 10 nM mirVana miRNA Mimic, negative control (#4464058, Thermo Fisher Scientific) was performed.

### 2.10 Statistical analysis

The analysis was performed using the GraphPad Prism software. Student’s *t*-test, ANOVA, and the Kruskal–Wallis test were used to assess the statistical significance. The data are presented as mean ± SEM. The differences were considered statistically significant at *p < 0.05, **p < 0.01, and ***p < 0.001.

## 3 Results

### 3.1 Tumor-derived ECs display different migration properties than healthy tissue–derived ECs

**FIGURE 1:**
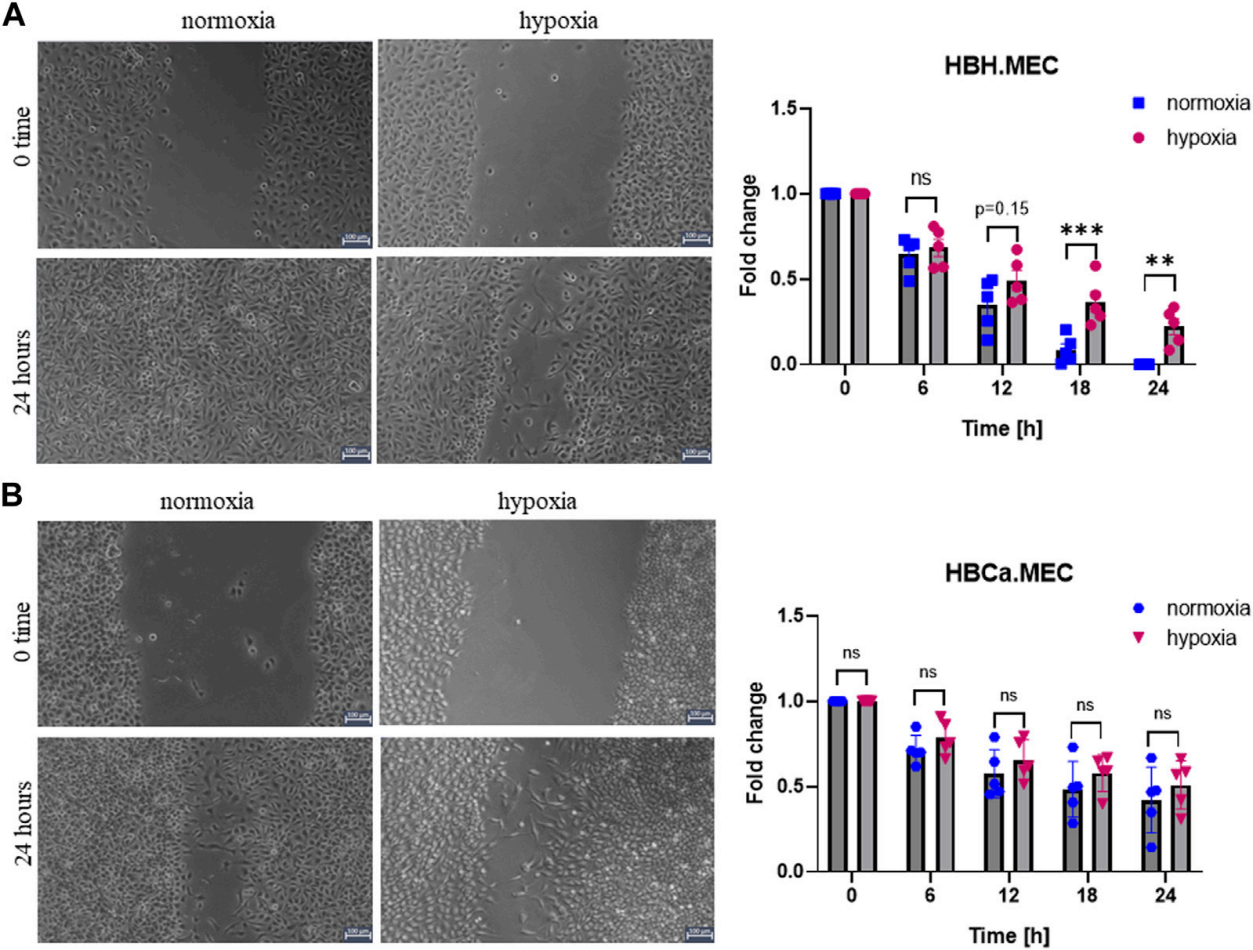
Hypoxia influences EC migration in wound healing assay performed with healthy and tumor-derived ECs. **(A)** Healthy breast tissue–derived ECs cultured in hypoxia and normoxia for 24 h, scratched cell monolayer, magnification ×5. *p < 0.05, **p < 0.01, and ***p < 0.001 in Student’s *t*-test vs. HBH.MEC normoxia. Data are reported as mean ± SEM (*n* = 5) normalized to HBH.MEC, normoxia = 1. **(B)** Breast tumor tissue–derived ECs cultured in hypoxia and normoxia for 24 h, scratched cell monolayer, magnification ×5. Data are reported as mean ± SEM (*n* = 5) normalized to HBCa.MEC, normoxia = 1.

To evaluate the EC migration properties triggered by hypoxia, we performed the functional wound healing test. The data show that healthy ECs migrate slower in hypoxia than in normoxia (Figure 1A), which was not observed in the case of tumor-derived ECs (Figure 1B). We observed no decreased migration ability upon hypoxia in this study.

### 3.2 Influence of EC tissue origin and biological state on global gene expression (HBCa.MEC vs*.* HBH.MEC, normoxia) and secretion of EndMT-related proteins

**FIGURE 2:**
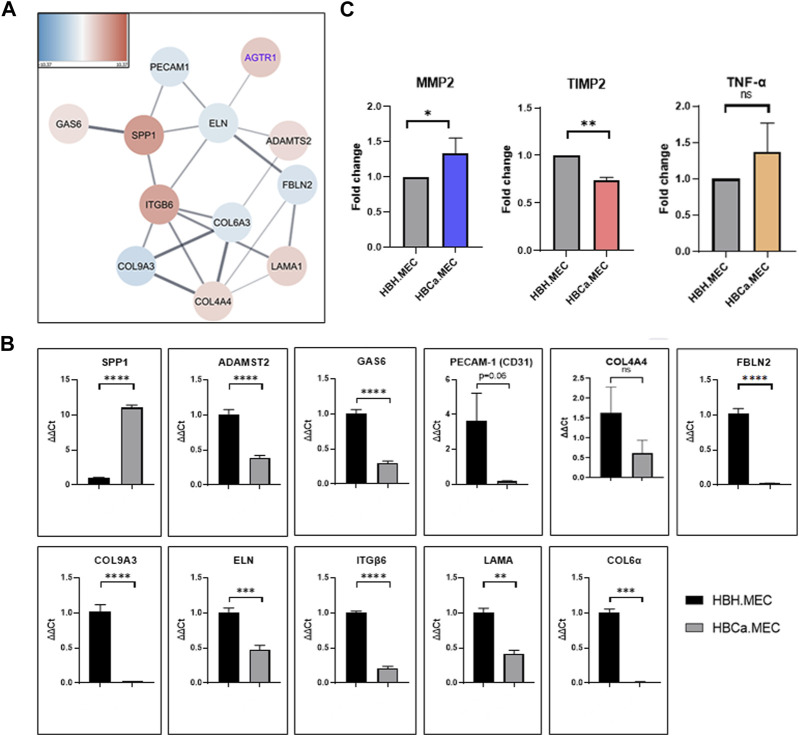
Tumor microenvironment's influence on the extracellular structure organization, transcriptome pattern, and protein secretion. **(A)** The functional enrichment protein network of the extracellular structure organization pattern. Functional enrichment networks were performed using Cytoscape software (v.3.8.0). **(B)** Gene expressions of *SPP1*, *COL4A4*, *PECAM1*, *ELN*, *FBLN2*, *COL6A3* and *COL9A3*, *ADAMST2*, *ITGB6*, *LAMA1*, and *GAS6* in HBH.MEC and HBCa.MEC were determined by quantitative RT-PCR (qRT-PCR); *PPIA* and *YWHAZ* act as quantitative internal controls. Data are reported as mean ± SEM (*n* = 3). **(C)** The level of proteins secreted to medium by HBH.MEC and HBCa.MEC after 48 h, measured by LEGENDplex beads, shown as fold changes of MFI. Data are reported as mean ± SEM (*n* = 3).

Next-generation sequencing (NGS) on healthy tissue–derived and pathological tissue–derived ECs from breast cancer has been published previously (Supplementary Figure S1) and revealed 350 upregulated genes and 396 downregulated genes (Wilkus et al., 2021). We used this data set for further analysis. The enrichment analysis based on the Gene Ontology pathway shows that the second and third most activated processes were related to extracellular matrix remodeling and structure, respectively (WEB-based Gene SeT AnaLysis Toolkit enrichment method: ORA; organism: *Homo sapiens*; enrichment categories: Gene Ontology biological_process). Among these, we found that the interaction network indicated the activity of fibronectin type 3 domain genes (Supplementary Figure S2A) and ECM-receptor interaction genes (Supplementary Figure S2B). The functional enrichment network of the extracellular structure organization pattern indicated the following genes to be upregulated: *SPP1*, *ITGB6*, *COL4A4*, *LAMA1*, *ADAMST2*, and *GAS6* and downregulated: *PECAM1*, *ELN*, *FBLN2*, *COL6A3*, and *COL9A3* (Figure 2A). In order to confirm the NGS data, we performed gene expression level validation by qPCR. The genes *SPP1*, *COL4A4*, *PECAM1*, *ELN*, *FBLN2*, *COL6A3*, and *COL9A3* present the same upregulated pattern in pathological cells vs. healthy ones, as the NGS data indicated. *ADAMST2*, *ITGB6*, *LAMA1*, and *GAS6* expressions were not confirmed as the NGS had indicated them as upregulated, while in qPCR, we observed downregulation (Figure 2B). Moreover, the secretions of MMP2, TIMP2, and TNF-α were measured. The secreted level of MMP2 was higher in pathological ECs than in healthy ECs. TNF-α was produced with no significant difference by both cell lines. The HBCa.MEC produced less TIMP2 than ECs derived from the healthy site of the breast (Figure 2C).

### 3.3 Hypoxia influences dysregulated miRNAs in tumor-derived ECs

**FIGURE 3:**
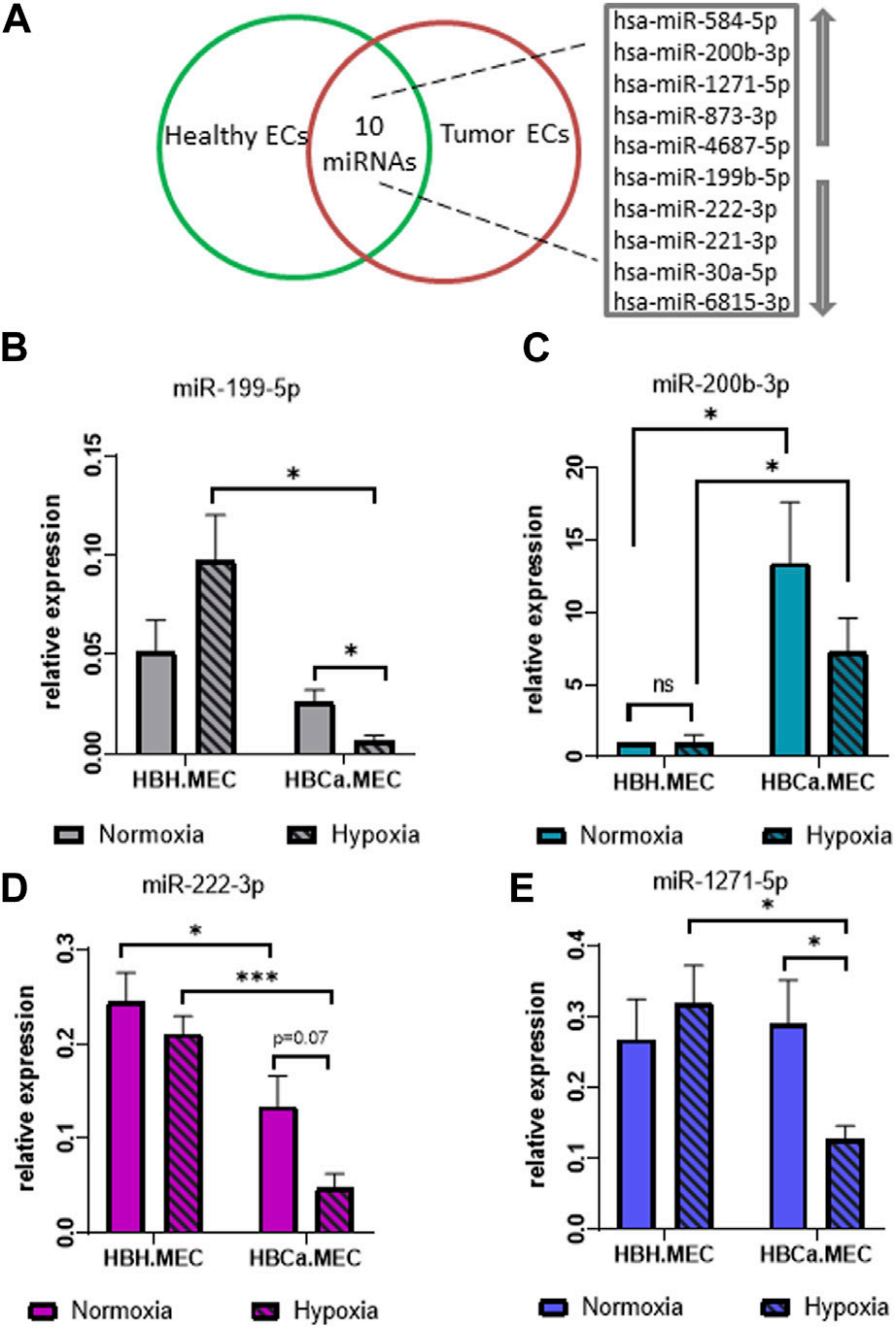
miRNAs most changed in breast tumor microenvironment and hypoxia-depended miRNA pattern in the organ-specific EC model. **(A)** List of the 10 most changed miRNAs between pathological and heathy breast endothelium. Arrows indicate upregulated and downregulated miRNAs. **(B)** miR-25-3p and miR-16-5p relative expressions of miR-199-5p in HBH.MEC and HBCa.MEC cultured in normoxic and hypoxic conditions. Data are reported as mean ± SEM (*n* = 3). **(C)** miR-25-3p and miR-16-5p relative expressions of miR-200b-3p in HBH.MEC and HBCa.MEC cultured in normoxic and hypoxic conditions. Data are reported as mean ± SEM (*n* = 5). Data were normalized to HBH.MEC = 1. **(D)** miR-25-3p and miR-16-5p relative expressions of miR-222-3p in HBH.MEC and HBCa.MEC cultured in normoxic and hypoxic conditions. Data are reported as mean ± SEM (*n* = 3). **(E)** miR-25-3p and miR-16-5p relative expressions of miR-1271-5p in HBH.MEC and HBCa.MEC cultured in normoxic and hypoxic conditions. Data are reported as mean ± SEM (*n* = 3).

The global analysis of microRNAs indicated 10 dysregulated microRNAs in tumor-derived ECs when compared to ECs obtained from the corresponding healthy tissue, which included five upregulated and five downregulated microRNAs as listed in Figure 3A. Among the upregulated microRNAs, we identified hsa-miR-584-5p, hsa-miR-200b-3p, hsa-miR-1271-5p, hsa-miR-873-3p, and hsa-miR-4687-5p, and among the main downregulated microRNAs were hsa-miR-199b-5p, hsa-miR-222-3p, hsa-miR-221-3p, hsa-miR-30a-5p, and hsa-miR-6815-3p (Figure 3A). In order to control the modifications in the miRNAs pattern in breast tumor–derived ECs, we validated the results of NGS by qPCR. The four most intensely changed miRNAs were hsa-miR-199b-5p, hsa-miR-222-3p and hsa-miR-200b-3p, hsa-miR-1271-5p being down- and upregulated, respectively. The validation data indicated the same pattern as the NGS results. miR-199b-5p and miR-222-3p were downregulated in HBCa.MEC when compared to HBH.MEC (Figures 3B, D). On the other hand, miR-200b-3p and miR-1271-5p were induced in pathological ECs vs. normal ones (Figures 3C, E). With the validation of NGS data by qPCR, we checked the miRNA level modulated not only by the EC origin but also by hypoxia. In normal tissue–derived ECs, the low pO_2_ did not influence the expressions of miR-222-3p, miR-200b-3p, and miR-1271-5p, whereas in HBCa.MEC, hypoxia had lowered their levels. miR-199b-5p is downregulated in response to hypoxia in tumor-derived ECs while being upregulated by hypoxia in normal tissue–derived ECs. miR-199b-5p downregulation is also enhanced in tumor-derived ECs when compared to healthy ECs in hypoxic conditions (Figure 3B). Taking into consideration the origin of ECs, miR-199b-5p, miR-222-3p, and miR-1271-5p were downregulated in tumor-derived ECs when compared to healthy ECs in hypoxia. Oppositely, miR-200b-3p upregulation was observed in tumor-derived ECs vs. normal tissue–derived ECs both in hypoxia as well as in normoxia (Figure 3C).

### 3.4 miRNA levels in organ-specific ECs related to tumor microenvironment influence

**FIGURE 4:**
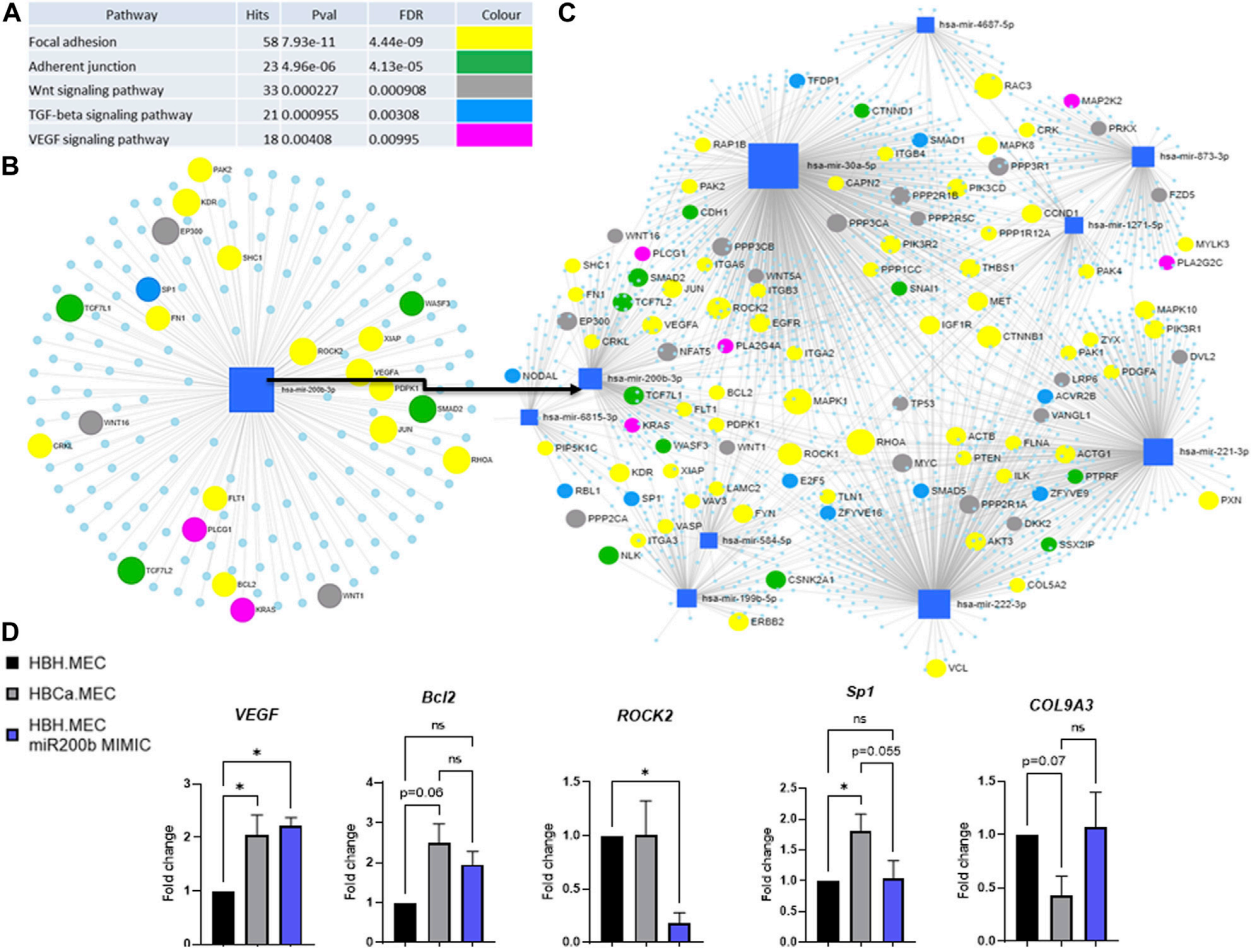
Predicted target genes of DEmiRNAs in the breast tumor microenvironment vs. healthy tissue. **(A)** Potentially deregulated signaling pathways for predicted target genes analyzed through susceptibility gene enrichment. **(B)** Pathway-related genes were regulated by miR-200b-3p. **(C)** Network of target genes, with marked genes involved in potentially dysregulated signaling pathways, predicted using miRNET software. **(D)** Regulation of target and EndMT genes by miR mimic. HBH.MEC and HBCa.MEC cells are negative controls, transfected with miR negative control. Data are reported as mean ± SEM (*n* = 3).

We identified the potential targets of these modified miRNAs, and Figure 4 presents the networks of the potential target genes, showing the number of targets for each miRNA (Figure 4C) and the signaling pathways related to the appointed target genes (Figure 4A). They are significantly involved not only in the focal adhesion and adherent cell junctions but also in the VEGF signaling pathway (Figure 4A). Accordingly to the last obtained data, we focused on hsa-miR-200b-3p-related genes and pathways related to pathological angiogenesis (Figure 4D) (Gregory et al., 2008). The HBH.MEC cells were transfected with miR-200b mimic and the level of the target genes was measured at the level of transcript and compared to cells, healthy and pathological, transfected with the negative control (Figure 4D). ROCK2 transcript was downregulated by exogenous miR-200b in healthy cells, and its level in the control, healthy and pathological cells, without treatment was similar. Bcl2 and Sp1 were not affected by mimic, however, the expression of these genes was increased in pathological cells when compared to HBH.MEC. The VEGF transcript was upregulated both in healthy cells treated by mimic and control pathological cells. COL9A3, not a direct target of miR-200b but a gene modulated by cell origin, was not affected by transfection.

## 4 Discussion

In our previously published data, we presented the distinctive properties of ECs from tumor and normal tissues in human breast cancer (Wilkus et al., 2021). In this work, we use this cell model to demonstrate how the tumor microenvironment influences the phenotype and biological properties of endothelial cells. We show that the tumor-derived ECs present distinct reactions from the corresponding microvascular endothelium in a non-pathologic microenvironment. For the future understanding of the evolution of pathology, such adaptations to the tumor microenvironment are highly significant. Indeed, we show here that the data obtained by a given EC type and origin cannot be transposed to other tissues and biological states. Presently, the work shows that the tumor microenvironment acts on EndMT induction and involves microRNA deregulation in breast cancer–derived ECs.

Functionally, the tumor tissue–derived ECs display distinct migration properties from normal ECs, which are not modified by hypoxia. Although endothelial cells originate from the same tissue, their response to hypoxia, mimicking the tumor microenvironment, is distinct and leads the cells to undertake separate and highly different responses. By questioning the differentially expressed genes of pathological endothelium vs. the healthy one, we did uncover new pathways involved in pathological angiogenesis and have demonstrated distinct gene activations in both types of cells. The analysis of the global gene expression identified not only several genes that are involved in extracellular matrix remodeling, such as collagens, laminins, fibronectin, and integrins, but also the metalloproteinase axis, as being deregulated in tumor-derived endothelial cells, together with genes leading the EndMT process. Cancer tissue–derived ECs are prone to better react to proangiogenic signals. This is confirmed and favored by their ability to putatively dedifferentiate as indicated by the selective engagement of the tumor tissue–derived ECs to undergo EndMT. This confirms our previously described results that suggested the pathological character of angiogenesis present in the endothelial cells from the breast cancer tissue (Wilkus et al., 2021).

Apart from the physiological microenvironment, many solid tumors express high levels of various ECM molecules like fibrillar collagens, fibronectin, elastin, and laminins (Mammoto et al., 2013). It is shown that triple-negative breast cancer and Her2 tumors present increased deposition of collagen and enhanced invasion with CAFs (Lochter and Bissell, 1995; Kular et al., 2014; Insua-Rodriguez and Oskarsson, 2016; Zheng et al., 2017; Henke et al., 2019). Up to now, the modified ECM molecules and their dysregulation in breast tumor endothelium are poorly described. Our data identified a few of these molecules to be deregulated in the pathological endothelium affecting the ECM pattern in a manner that is distinct from healthy tissues, indicating their “mesenchymal” phenotype and tumor-related plasticity. Zhao et al. (2018) indicated that *SSP1* expression increased in solid tumors; here, we show that this is also operated by the endothelial cells in pathological tissues as opposed to healthy breast ECs. This correlates with the data presented by Rudland et al. (2002), who have presented the same tendency but in breast tumor cells. Moreover, our data present the overexpression of integrin *ITGB6* in pathological ECs, which confirms that the previously described processes require tissue remodeling (Sheppard et al., 1990). *ITGB6*, one of the β6 subunit of the integrin αvβ6, is highly expressed on activated endothelial cells, newly formed vessels, and on some tumor cells (Caiado et al., 2011). TGF-β1 works as part of a positive feedback loop promoting the expression of *ITGB6* (Sheppard et al., 1990). Additionally, secreted factors such as MMP2 and TIMP2, which contribute to the shaping of ECM, show a pattern described as typical for EndMT. Increased activity of MMPs is described as the driving feature of EndMT (Evrard et al., 2016), as recombinant MMP, similar to TGF-β, can induce EndMT *in vitro*. Our data from the miRNA analysis also contribute to indicate the TGF-β signaling pathway's involvement and association with EndMT (Zhao et al., 2016).

In the present study, we have shown that mature, organ-specific ECs obtained from the mammary gland, tumor site and healthy organ, vary in miRNA expression. The analysis of the potentially most influenced pathways shows, among others, focal adhesion. It confirms the results obtained by Natale et al. (2019), who demonstrated that the key factor of cellular morphology regulation is focal adhesion clustering that subsequently drives cytoskeletal organization.

Triple‐negative breast cancer, the source of our EC model, is characterized by high levels of miR‐200b, what correlates with a poor patient outcome (Sanchez-Cid et al., 2017). This may be explained by the fact that miR‐200 is highly expressed in basal‐like metastatic cancer cells of triple‐negative breast cancer which is the most aggressive group of breast cancer (Sanchez-Cid et al., 2017). The microRNA-200 (miR-200) family, which regulates mesenchymal to epithelial transition, is enriched in the serum of patients with metastatic cancers (Le et al., 2014; Yao et al., 2015). Our data indicate that miR-200b was upregulated in pathological ECs vs. healthy endothelium upon normoxia. As some studies have indicated, miR-200b silences angiogenesis and is correlated with the metastatic cascade in breast tumor, and we present that mir-200b-3p is also deregulated in endothelial cells derived from this pathological tissue (Amorim et al., 2019; Chen et al., 2022) The bioinformatics analysis shows that VEGFA and KDR1 (VEGFA receptor) may be mir-200b-3p target genes. In our model, this effect was not observed at the transcript level as both miR-200b that is high in cancer tissue–derived endothelial cells and miR mimic-treated healthy cells were characterized by VEGF mRNA. However, in our previously published experiments, cancer-derived ECs produced less VEGF measured at the level of proteins (Wilkus et al., 2021). It is quite common that mRNA and protein levels do not correspond upon miR regulation, therefore the modulation of VEGF might have occurred through the inhibition of translation like how microRNAs often operate (Pillai et al., 2005), than through transcript degradation (Choi et al., 2011). Similarly, the protein level of KDR1 (FLK-1; CD309) was lower in pathological endothelium than in healthy ECs, which could be a consequence of mir-200b-3p regulation (Wilkus et al., 2021). Angiogenic potential was lower in cancer ECs vs. healthy ones upon normoxia that is correlated with the upregulation of mir-200b-3p. These findings corroborate the data presented by Sinha et al. (2015), who claim that miR-200b silences several angiogenic growth factors and their receptors by directly targeting their mRNA transcripts. Our data confirm that the pathological phenotype of tumor-derived ECs can be regulated by this miR, which is strongly affected by hypoxia. We also tested other miR-200b targets such as ROCK2, Bcl2, and SP1 and only the first was downregulated by the mimic, as observed by others (Peng et al., 2013). However, in the case of miR-200b, highly expressing in cancer cells (Peng et al., 2013), ROCK2 was not downregulated, which additionally points to deregulated responses of pathological cells. Moreover, Bcl2 and Sp1, which are miR-200b targets, were increased in cancer-derived endothelial cells when compared with healthy ECs that are characterized by very low miR-200b expression. It is known that microRNAs can not only downregulate their target genes but also increase their expressions. Initially, this was particularly observed in quiescent, G0 cells (Truesdell et al., 2012), and now it is known that the outcome of microRNA regulation highly depends on the cell type and biological state (reviewed by Valinezhad Orang et al., 2014). Therefore, the pathological ECs present not only a deregulated microRNA pattern but also an altered response to their regulatory effects (Valinezhad Orang et al., 2014).

In addition, other microRNAs were found characteristic for the present pathological ECs. The effect of miR-199b-5p on tumor angiogenesis remains unclear. It has been shown that low miR-199b-5p levels are associated with a poor prognosis of breast cancer (Fang et al., 2013). In our model, we report that the level of this miRNA is indeed lower in breast tumor–derived ECs than in healthy endothelium from the same organ that correlates with Fang et al.’s (2013) findings (Le et al., 2014).

The miR-221/222 cluster not only controls development and differentiation of ECs but also inhibits their proangiogenic activation, proliferation, and migration. miR-221/222 is primarily implicated in maintaining endothelial integrity and supporting quiescent EC phenotype (Chistiakov et al., 2015). In our study, this miRNA was downregulated in the pathological endothelium as compared to the normal ones and confirms the above described features. Our previously obtained data (Wilkus et al., 2021) have indicated that tumor-derived ECs proliferate slower and have lower pro-angiogenic response in normoxia than healthy ECs.

To date, for the first time we present changed hsa-miR-1271-5p in breast tumor–derived endothelial cells. hsa-miR-1271-5p is described as downregulated in breast cancer tissues when compared to normal breast tissues (Du and Liu, 2018), and we have shown that in hypoxic tumor microenvironment, pathological ECs also show lower level of hsa-miR-1271-5p than do healthy ones, which highlights the key effect of microenvironmental conditions.

## 5 Limitations

The cells were obtained from one donor and immortalized with a previously designed and patented protocol (Kieda et al., 2002). The isolation of ECs from numerous donors with breast cancer would be important to support the present demonstration and validate on a wide range of breast tumor cases. In addition, such models of immortalized cell lines provide ECs with a stabilized phenotype, allowing continuous culture conditions of a well-characterized set of cell lines (Wilkus et al., 2021).

## 6 Conclusion

Taking all together, our data indicate that breast endothelial cells differ phenotypically and functionally depending on their origin: healthy and tumor tissue. It highlights the modulatory role of the tumor microenvironment which, apart from disturbing angiogenesis, influences the phenotypic changes in pathological ECs inducing EndMT. Among the most changed miRNAs in the pathological endothelium when compared to the healthy one, miR-200b-3p has the potential to regulate the angiogenic response; however, its activity is altered by the cell origin.

## Data Availability

The data presented in the study are deposited in the GEO repository, accession number GSE224147 and GSE179509 [Wilkus et al., 2021].

## Supplementary material

SUPPLEMENTARY FIGURE S1 Differences in the transcriptome profiles of tumor-derived ECs and healthy ECs. Functional enrichment network performed using Cytoscape software (v.3.8.0).

SUPPLEMENTARY FIGURE S2 Differences in the transcriptome profiles of tumor-derived ECs and healthy tissue–derived ECs. **(A)** The functional enrichment network of fibronectin type 3 domain, type III, and type III superfamily. **(B)** The functional enrichment network of the ECM-receptor interaction. NGS data have been deposited in NCBI’s Gene Expression Omnibus (Edgar et al., 2002) and are accessible through GEO Series accession number GSE224147 (https://www.ncbi.nlm.nih.gov/geo/query/acc.cgi?acc=GSE224147).
