# Supplementary figures and images for: Microenvironment commits breast tumor ECs to dedifferentiation by micro-RNA-200-b-3p regulation and extracellular matrix remodeling
Source: Front Cell Dev Biol. 2023 May 16;11:1125077. doi: 10.3389/fcell.2023.1125077 (PMC10229062; doi:10.3389/fcell.2023.1125077)

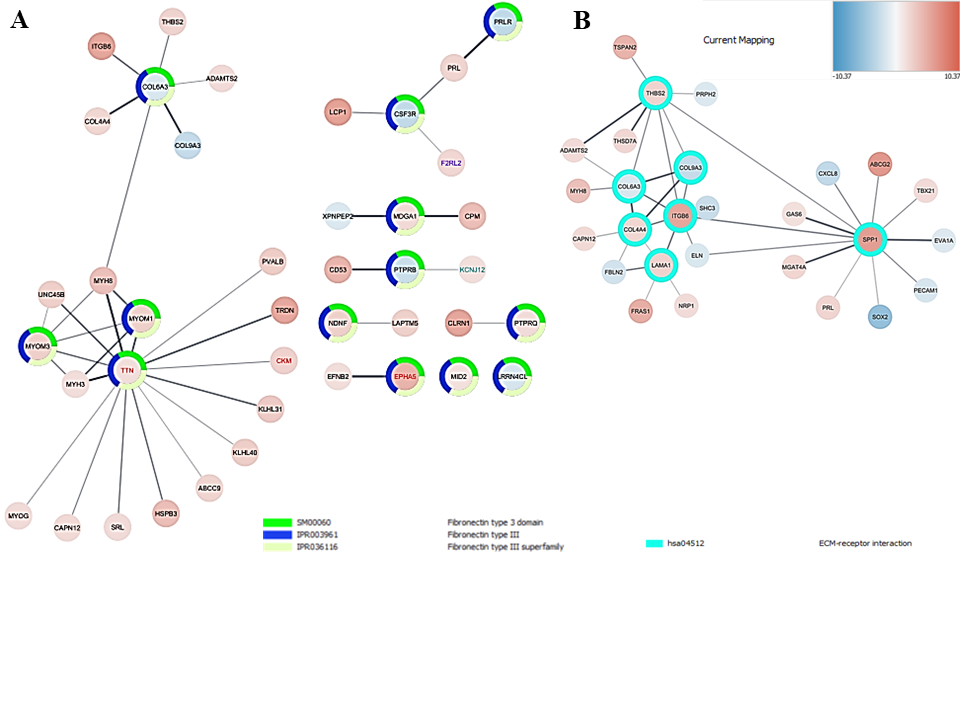

Supplement: Supplementary file 1 [file Image2.TIF]

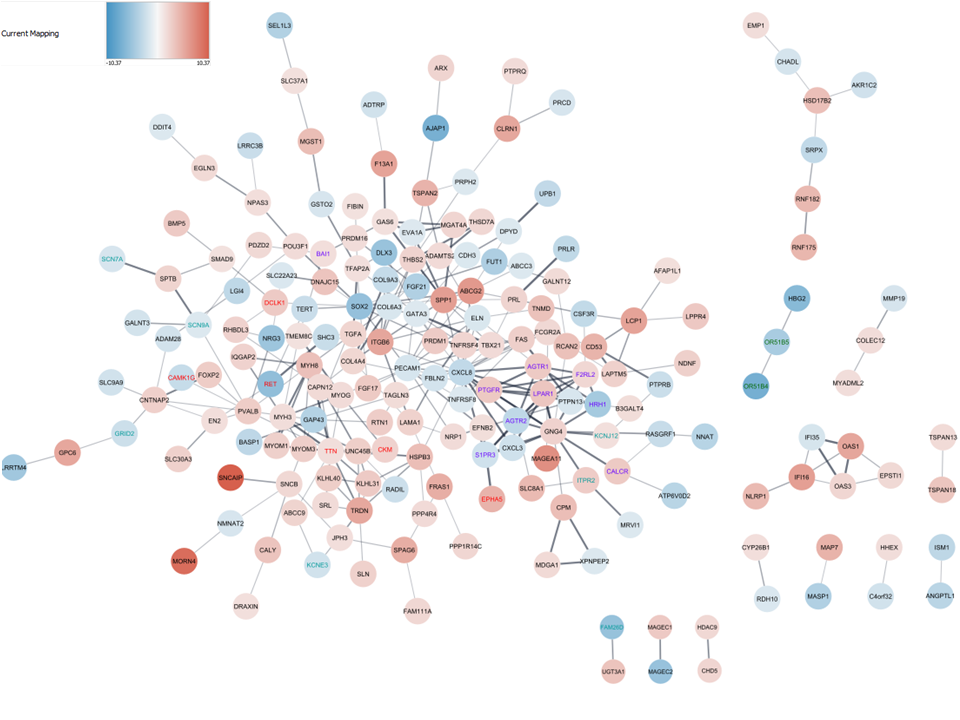

Supplement: Supplementary file 2 [file Image1.TIF]
